# Transcriptomic Study of *Nicotiana tabacum* Treated with the Bacterial Protein CspD Reveals Some Specific Abiotic Stress Responses

**DOI:** 10.3390/ijms252313015

**Published:** 2024-12-03

**Authors:** Denis Erokhin, Diana Baranova, Ksenia Sergeeva, Tatiana Pasechnik, Larisa Shcherbakova, Natalia Statsyuk, Vitaly Dzhavakhiya

**Affiliations:** All-Russian Research Institute of Phytopathology, 143050 Bolshie Vyazemy, Russia; erokhin.denis.v@gmail.com (D.E.); dzhavakhiya@yahoo.com (V.D.)

**Keywords:** plant resistance, defense elicitor, bacterial cold-shock protein, CspD, ISR, abiotic stress, light response, Cu-Zn superoxide dismutase, RNA-seq

## Abstract

The ability of a cold-shock protein CspD from *Bacillus thuringiensis* to protect both dicots and monocots against various pathogens is well confirmed under both greenhouse and field conditions; however, the molecular basis of this phenomenon at the transcriptomic level still remains unexplored. Expression profiles of some marker genes associated with SAR/ISR nonspecific resistance pathways and ROS scavengers were examined in CspD-treated *Nicotiana tabacum* plants, and the RNA-seq analysis of CspD-treated plants was first carried out. The ISR markers PDF1.2 and PR4 were overexpressed locally in treated tobacco leaves with the maximum 2.4- and 5.7-fold change, respectively, reached 12 h after the leaf treatment with CspD; PDF1.2 was also up-regulated 4.8-fold four days after the inoculation of treated plants with TMV. The ROS scavenger analysis demonstrated overexpression of Cu-Zn superoxide dismutase in both treated (with the maximum 5.4-fold change observed 6 h after the treatment) leaves and leaves from the upper tier (“system” leaves, 6.5-fold change observed 4 days after the treatment). The ROS assay confirmed endogenous accumulation of superoxide in CspD-treated leaves 6 and 24 h after the treatment. An in silico comparative study of *Arabidopsis* orthologs of highly up-regulated tobacco genes induced by CspD with *Arabidopsis* genes activated by some other molecular patterns revealed the specific CspD-induced expression of Cu-Zn superoxide dismutase and some other genes associated with light and cold responses. This study may contribute to a better understanding of cross-talking between abiotic stress and nonspecific immunity in plants.

## 1. Introduction

The application of defense elicitors to establish the nonspecific plant resistance to biotic stress is based on the use of biogenic compounds (i.e., compounds produced by living organisms) and helps to maintain more sustainable agriculture compared to using pesticides only [1,2]. Contrary to synthetic inducers of plant resistance to diseases, biogenic elicitors, being ubiquitous in biomes and possessing the higher rate of biodegradation compared to xenobiotics, do not result in accumulation of toxic residues, thus providing less xenobiotic pressure on the environment. The application of elicitors relies on the hormone-regulated mechanisms of innate plant immunity. In many cases, elicitors activate one or both of the mainstream pathways, leading to the development of systemic resistance in the entire plant: salicylic acid (SA)-dependent systemic acquired resistance (SAR) [3]; and jasmonic acid (JA)- and ethylene (ET)-dependent induced systemic resistance (ISR) [4].

The majority of the currently known biogenic elicitors are microbial metabolites produced by fungi, bacteria, viruses, or oomycetes. They act as elicitors of plant immunity when interacting with a host plant and are generally called microbe-associated molecular patterns (MAMPs) or (for those originating from pathogenic microorganisms) pathogen-associated molecular patterns (PAMPs). MAMPs/PAMPs may have variable structures; however, the most common of them represent oligosaccharides, glycopeptides, and protein-based substances [1]. For example, β-glucans [5,6,7], chitin [8,9], and chitosan [10,11] are common oligosaccharide elicitors of fungal origin. BcGs1 is a glycopeptide elicitor from *Botrytis cinerea* [12]. The examples of protein-based elicitors of the bacterial origin include the flg22 peptide (a part of bacterial flagellin) [13,14,15,16,17]; elf18 peptide (a part of the Tu elongation factor) [18]; csp22 and csp15, representing a consensus sequence of the RNP-1 RNA-binding motif of cold-shock proteins [19]; and harpin proteins, which are components of the bacterial transport system III [20,21,22]. A special interest in protein elicitors is caused by the added benefits of the robust recombinant protein technology [23] to produce active ingredients for commercial use. Messenger^®^ [24] and ATaiLing [25] are probably the most well-known examples of successfully commercialized protein elicitors.

In previous years, a bacterial cold-shock protein possessing the ability to elicit early immune reactions in both dicotyledonous and monocotyledonous plants was obtained from the cell extracts of *Bacillus thuringiensis*. The structures of this elicitor protein and the corresponding encoding gene were identified and patented [26,27]. The sequence of the encoding gene called CspD (cold shock protein D) was deposited in the GenBank with the accession number AY272058, thus supplementing the data about the CspA and CspB proteins from *B. thuringiensis* available at that time. The obtained data were subsequently confirmed by another research group, who discovered that the bacterial cold-shock protein from *Micrococcus lysodeikticus* (*Staphilococcus aureus*) could work as a nonspecific elicitor of defense reactions in plants from the family *Solanaceae* but was inactive towards rice, cucumber, and some other plants [19]. The alignment of the amino acid sequence of this protein with 22 cold-shock proteins from other bacteria identified a consensus sequence consisting of 22 amino acids and called csp22, which represented an RNA-binding motif RNP-1 of bacterial cold-shock proteins. The protecting activity of a synthetic csp22 peptide in *Nicotiana tabacum* exceeded that of the original cold-shock protein from *M. lysodeikticus*. The further reduction of the csp22 sequence revealed 15-amino acid region called csp15, which possessed almost the same protective activity as csp22 and was therefore considered to be the minimum functional core of bacterial cold-shock proteins possessing eliciting activity. Pattern-recognition receptors for csp22 were identified only in plants from the family *Solanaceae* and included the receptor-like protein NbCSPR from *Nicotiana benthamiana* [28] and its homologue, a CORE receptor from *S. lycopersicum* encoding leucine-rich repeat receptor-like kinase with structural resemblance to the EF-Tu receptor [29]. Studies investigating the molecular mechanisms behind the plant resistance induction by bacterial cold-shock proteins have primarily focused on elucidating the functional structure of these proteins and their immune recognition in plants. However, little is known about the mechanisms that operate downstream of their detection by plant cell receptors. Moreover, compared to other common MAMPs/PAMPs, the studies of bacterial cold-shock proteins possessing eliciting activity are characterized by a notable absence of omics investigations, which could provide a comprehensive understanding of plant responses to such elicitor stimuli. The only exception is the metabolomics analysis of a csp22 challenge in *Solanum lycopersicum* [30].

The known biochemical effects of bacterial cold-shock proteins eliciting plant immune responses include stimulation of ethylene production, which is associated with the development of the ISR-type nonspecific resistance in tomato plants treated with csp15 and csp22 [19,29]; additionally, csp15 was shown to induce an oxidative burst in tomato plants [19] and in certain csp22-treated *Rutaceae* plant species [31].

Assessing the expression profiles of some key marker genes related to the nonspecific resistance and utilization of reactive oxygen species (ROS) can be considered as a valuable starting point for the presented study, which is aimed at investigating the response of a tobacco transcriptome to the CspD treatment. The performed RNA-seq analysis represents the first transcriptomic study exploring plant responses to the treatment with bacterial cold-shock proteins with confirmed eliciting activity. The results of this analysis were subsequently used for an in silico comparative analysis against the transcriptomic data related to some individual and complex molecular patterns, including flg22 [32], elf28 [33], harpin [34], oligogalacturonides (OGs) [32], lipopolysaccharides (LPS) [34], and bacterial outer membrane vesicles (OMVs) [35].

## 2. Results

### 2.1. Differential Gene Expression Analysis of SAR/ISR Marker Genes

Several tests were conducted to identify the type of nonspecific resistance. In the first experiment, the dynamics of local expression of specific marker genes associated with SAR (*pal*, *ics1*, *pr1a*) and ISR (*pdf1.2*, *chiB*, *pr3*, *pr4*) were examined in CspD-treated third-layer (“local”) tobacco leaves compared to control samples. The second experiment assessed the systemic expression of SAR/ISR marker genes in the “system” (fourth-tier) tobacco leaves four days after the initial treatment of “local” leaves with CspD (compared to the control samples). In the third experiment, the local expression of SAR/ISR marker genes was investigated five days after the treatment of tobacco leaves with CspD followed by the next-day inoculation with tobacco mosaic virus (TMV) and compared to the control samples.

**Figure 1:**
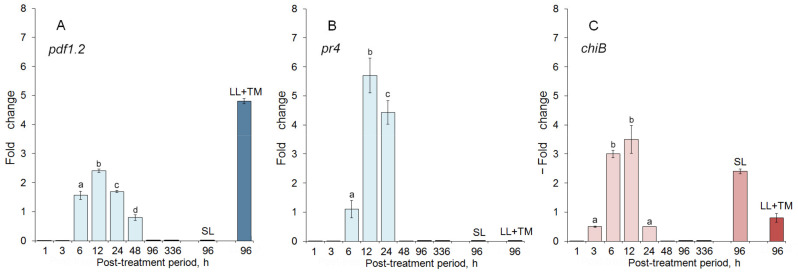
DEG analysis of altered SAR/ISR marker genes: (**A**) *pdf1.2*; (**B**) *pr4*; (**C**) *chiB*. Blue and red colors indicate up- and down-regulation of the corresponding gene, respectively. The series of time points marked in pale color on the left side of each diagram indicate the dynamics of gene expression in the “local” tobacco leaves after exposure to CspD. “SL” indicates the experiment on “system” leaves. “LL + TMV” indicates the “CspD treatment + TMV post-inoculation” test. Two independent experiments were performed, each including 10–12 biological replicates. Different letters above the bars indicate statistically significant difference (*p* ≤ 0.05) between the values of fold changes.

The DEG analysis of SAR/ISR marker genes revealed up-regulation of the expression of the *pdf1.2* gene, a JA/ET-responsive aggregator representing an ISR marker gene (Figure 1A). The maximum fold change for this gene observed in the “local” leaves 12 h after the treatment with CspD was 2.4; in the case of the CspD treatment followed by the inoculation with TMV, it reached 4.8. No changes in the expression of the *pdf1.2* gene were observed in the “system” leaves.

The up-regulation of the pathogenesis-related (PR) *pr4* gene (another ISR marker) reached the maximum (5.7-fold change) 12 h after the treatment with CspD (Figure 1B). Interestingly, the expression of another PR-ISR marker, a *chiB* gene, was down-regulated with the minimum fold change equal to 0.25 at the same time point (Figure 1C). In “system” leaves, the minimum fold change was 2.4, while the combined CspD-TMV treatment of “local” leaves resulted in almost no changes in its expression level (Figure 1C). The expression of SAR-associated genes was either unchanged or down-regulated at some time points. The complete expression profiles of the studied genes are provided in the Supplementary Materials (Table S1).

The up-regulation of expression of the ISR marker genes *pdf1.2* and *pr4* after the CspD treatment and the absence of the up-regulation of expression of SAR-associated genes suggests the ISR type of immune response in tobacco induced by the CspD treatment.

### 2.2. Differential Gene Expression Analysis of ROS Scavenging Genes

The expression of ROS scavenging genes, namely, superoxide dismutase (*sod*), catalase (*cat*), and ascorbate peroxidase (*apx*), representing the most common antioxidant enzymes in tobacco, was analyzed under the same conditions as for SAR/ISR marker genes described in Section 2.1.

**Figure 2:**
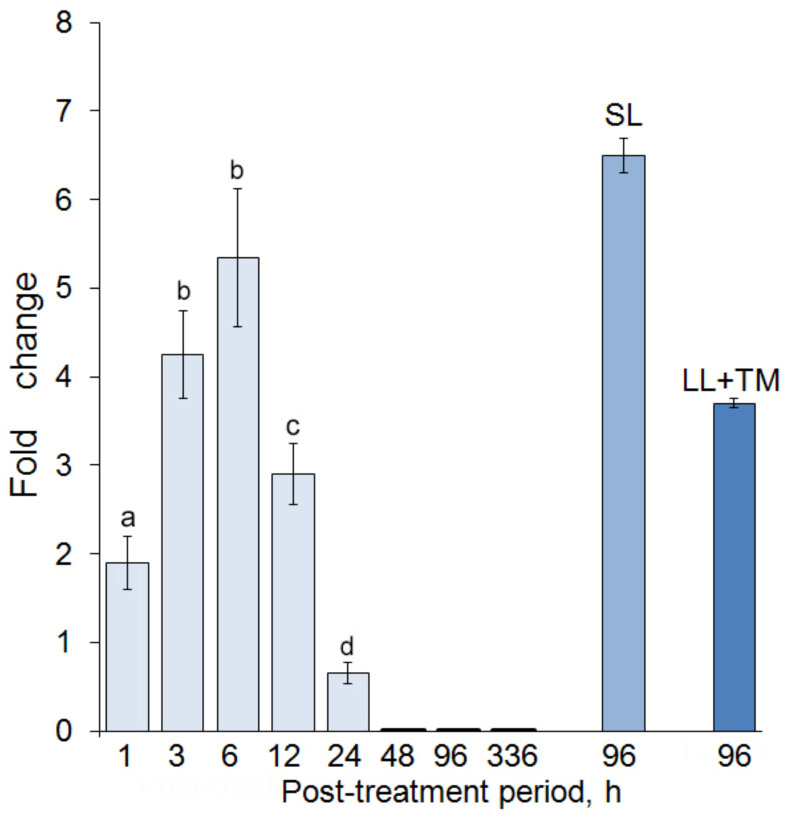
Changes in the expression level of a superoxide dismutase (*sod*) gene in tobacco leaves caused by the CspD and CspD + TMV challenges. The series of time points marked in pale color on the left side of the diagram shows the *sod* expression dynamics in the “local” tobacco leaves after their treatment with CspD. “SL” indicates the experiment on “system” leaves; “LL + TMV” indicates the “CspD treatment + TMV post-inoculation” test. Two independent experiments were performed, each including 10–12 biological replicates. Different letters above the bars indicate statistically significant difference (*p* ≤ 0.05) between the values of fold changes.

The DEG analysis revealed that only the *sod* gene was up-regulated after the leaf treatment with CspD (Figure 2). The fold change for the *sod* expression in the “local” leaves reached the maximum (5.4) 6 h after the treatment. Moreover, the *sod* gene was up-regulated 6.5-fold in the “system” leaves 4 days after the CspD challenge. In the case of the TMV post-inoculation, expression of this gene showed a 3.7-fold increase, thus proving its involvement into both priming and further protection against the TMV challenge. The expression of the *cat* gene showed a 1.9- and 1.5-fold decrease 3 and 6 h after the CspD treatment of “local” leaves, respectively. Expression of the *apx* gene showed no significant changes under all treatment conditions. The complete expression profiles of the studied genes are provided in the Supplementary Materials (Table S1).

The up-regulation of a superoxide dismutase gene revealed under all studied conditions demonstrates an important role of this enzyme in the CspD-induced immune response in tobacco.

### 2.3. RNA-Seq Anaysis of CspD-Induced Response in Tobacco

To study the transcriptomic response of tobacco to the CspD challenge, tobacco leaves were treated with CspD or water as the control. A total of 2301 and 861 DEGs were found to be significantly (|log_2_FC| ≥ 1, adjusted *p*-value < 0.05) up- or down-regulated, respectively, in response to the treatment (Supplementary Materials, Table S2). The log_2_FC parameter measuring the level of gene expression ranged from the maximum value of 5.32 (LOC107788102), which corresponded to a 39.9-fold change, to −5.58 (LOC107785603), which corresponded to a 0.02-fold change.

The top-40 up-regulated DEGs included a number of abiotic stress-responsive genes, such as the ERD6-like sugar transporter (LOC107788102) or E3 ubiquitin–protein ligase RGLG1-like (LOC107806658), and defense-related elements, such as WRKY transcription factors (LOC107829848, LOC107832371), PEARLI1-like lipid transfer protein 1 (LOC107792217), or probable disease resistance protein RPP1 (LOC107815900). Functions of the majority of the top-30 down-regulated genes are unknown; however, some of these genes are annotated as proteinase inhibitors (LOC107827891, LOC107794464, LOC107771920, LOC107790557).

To determine whether biological processes related to the plant immunity were positively affected by the CspD treatment of tobacco leaves, over-representation analysis (ORA) was performed for the initial list of 2301 DEGs with log_2_FC ≥ 1, 911 DEGs with log_2_FC ≥ 1.5, and 293 DEGs with log_2_FC ≥ 2, which represented up-regulated genes with the low, medium, and high levels of expression, respectively (see Supplementary Materials, Table S3). The ORA test with the FDR threshold value of 0.05 revealed no statistically significant results for DEGs with a high expression level (log_2_FC ≥ 2) but showed a wide range of biological processes for the entire list of up-regulated DEGs, which was found to be difficult to interpret in terms of their biological meaning. However, medium-expressed DEGs with log_2_FC ≥ 1.5 demonstrated a significant enrichment with defense-related GO terms.

The presence of defense-related genes in the list of top-40 up-regulated genes, as well as the enrichment of medium expressed genes with defense-related GO terms, confirms the elicitor activity of CspD.

### 2.4. ORA Tests of the Arabidopsis Orthologs of High-Expression Tobacco DEGs

**Figure 3:**
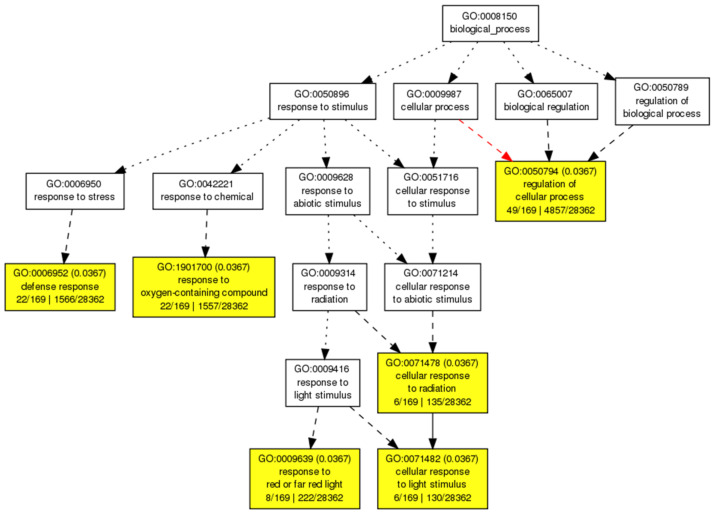
The tree of GO terms for biological processes obtained via the enrichment analysis of available *Arabidopsis* orthologs of highly expressed DEGs of CspD-treated tobacco leaves using an AgriGO SEA web tool (https://systemsbiology.cau.edu.cn/agriGOv2/c_SEA.php, accessed on 29 August 2024). The terms with FDR < 0.05 are marked in yellow. Arrows represent connections between different GO terms. Black and red arrows indicate “is-a” and “part-of” relationships, respectively. Solid, dashed, and dotted arrows represent two, one, and zero enriched terms at both ends connected by the arrow, respectively. The rank direction of the tree graph is set to from top to bottom.

To identify the putative biological processes related to the high-expression tobacco DEGs (log_2_FC ≥ 2), where the direct ORA test did not show statistically significant results, the additional ORA tests were conducted for the available *Arabidopsis thaliana* orthologs of high-expression tobacco DEGs (see Supplementary Materials, Table S4; Files S1 and S2). The performed analysis revealed the following over-represented GO terms (Figure 3): “defense response” (GO: 0006952), “response to oxygen-containing compound” (GO: 1901700), “response to red or far red light” (GO: 0009639), “cellular response to light stimulus” (GO: 0071482), and “regulation of cellular processes” (GO: 0050794). Thus, the obtained results demonstrated that, along with the general GO term “regulation of cellular processes” and the expected GO term “defense response”, such GO terms as the “response to red or far red light” and “response to oxygen-containing compound”, both related to abiotic stress response, may also play a role in biological processes triggered by the CspD treatment.

### 2.5. Comparative Study of the Results of the Defense Response Induction in Arabidopsis by CspD, flg22, OGs, and OMVs

**Figure 4:**
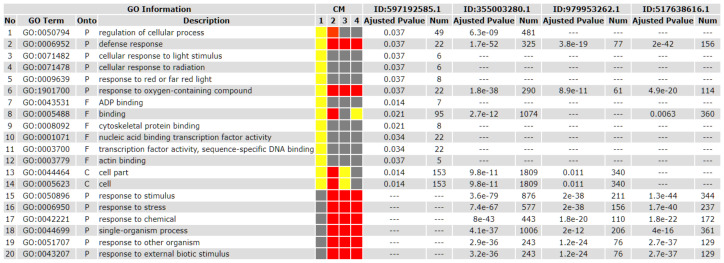
Comparison of the enriched GO terms for highly expressed *Arabidopsis* orthologs of DEGs of CspD-treated tobacco leaves with other common MAMPs. **Top**: reduced range of compared elicitors, from left to right: CspD, flg22, OGs, and OMVs. **Bottom**: full range of compared elicitors, from left to right: CspD, flg22, OGs, and OMVs. A colorful model (CM) represents the SEACOMPARE relative statistical significance of enriched GO terms in four elicitor treatments (“1” for CspD, “2” for flg22, “3” for OGs, “4” for OMVs); highly significant results are indicated in red, moderately significant results are indicated in yellow, and the results which did not pass the “FDR < 0.05” threshold are indicated in grey.

To examine whether the CspD treatment is able to induce any specific biological processes, a comparison of enriched GO terms for *Arabidopsis* orthologs of highly expressed (log_2_FC ≥ 2) DEGs of CspD-treated tobacco leaves vs. a full range of up-regulated (log_2_FC ≥ 1) DEGs generated by the treatment of *Arabidopsis* by flg22, Ogs, and OMVs molecular patterns was carried out. The performed analysis showed that CspD shared GO terms related to the plant defense response (GO: 0006952) to other elicitors; in addition, both web tools used for the analysis generated the same unique GO terms (Figure 4), all related to abiotic stress responses such as “cellular response to light stimulus” (GO: 0071482), “cellular response to radiation” (GO: 0071478), and “response to red and far red light” (GO: 0009639).

Three unique CspD-induced GO terms consisted of ten non-intersecting genes (Supplementary Materials, Table S5): Cu-Zn superoxide dismutase 2, GIGANTEA, ELF3, ANNAT5, JAC1, PHYTOCHROME E, PIL5, GIBBERILLIN 2-OXIDASE 6, ATP-dependent helicase, and ARGONAUTE 1.

Thus, the obtained results suggest that response to light may be a specific induction outcome of the CspD treatment.

### 2.6. RNA-Seq Validation

To evaluate the validity of RNA-seq results, the expression of ten up-regulated genes, including some of those related to the light stimulus response, was examined via quantitative PCR (qPCR) using specific primers (Supplementary Materials, Table S6) as described in Materials and Methods (Section 4.3). All of the tested genes demonstrated the same pattern as in the RNA-seq study and were notably up-regulated compared to the control with the only exception of Cu-Zn superoxide dismutase 2 (CSD2), which did not show significant changes in its expression level, apart from a very similar izoenzyme Cu-Zn superoxide dismutase 1 (CSD1), which was used in the evaluation of the differential expression of ROS scavenging genes. CSD2 might be misinterpreted by the sequencing platform due to its very high sequence similarity with CSD1, so including CSD2 to the DEG list may be considered as a sequencing error. With this exception, qPCR experiments confirmed the validity of RNA-Seq results.

### 2.7. ROS Analysis Indicated Accumulation of Endogenous Superoxide After the CspD Treatment

**Figure 5:**
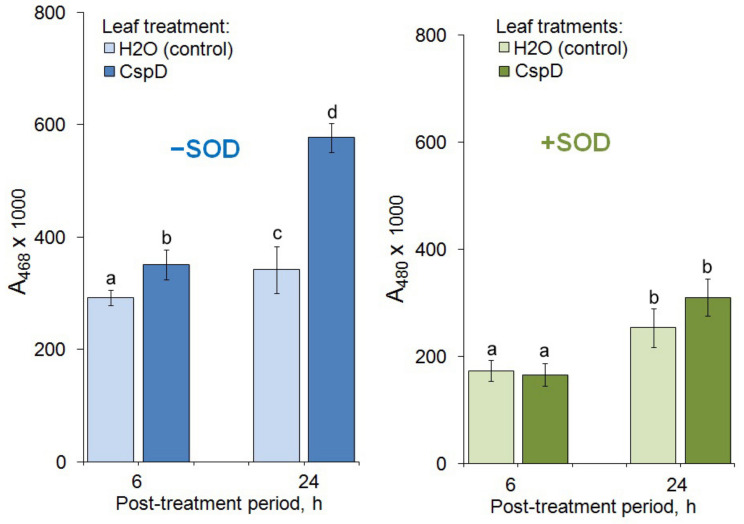
Adrenaline oxidation in diffusates of CspD-treated tobacco leaves in the absence (**left**) and in the presence (**right**) of SOD added into the reaction mixture. Two independent experiments were performed, each including three biological replicates. Different letters above the bars indicate a significant difference at *p* ≤ 0.05, while the same letters show statistically insignificant difference.

The ROS assay was used for the biochemical validation of the gene expression data. Samples of “local” leaves prepared 6 and 24 h after the CspD treatment showed a 20 and 69% increase in absorbance associated with the adrenaline oxidation by ROS, respectively, compared to the samples treated with water (Figure 5, left part). Superoxide dismutase addition to the reaction mixture did not reveal any significant difference between the treated and control samples that confirmed the endogenous accumulation of superoxide after the CspD treatment (Figure 5, right part).

## 3. Discussion

Profiling the elicitor-triggered non-specific resistance can help to understand the elicitor mode of action and may be valuable for combining elicitors with different resistance profiles to benefit from either way of protection. The lack of changes in the expression level of SAR-associated genes suggests that the SA-dependent pathway is not involved in the immune response of tobacco plants to CspD. The up-regulated expression of ET/JA-regulated pathogenesis-related (PR) genes after the CspD treatment and the *pdf1.2* gene after the further inoculation with TMV in local tobacco leaves advocates for the ISR-type of nonspecific resistance induced by the CspD treatment. However, a significant down-regulation of the *chiB* gene (another ET/JA-regulated PR gene) does not support the CspD-triggered ISR. Thus, the origin of CspD (growth-stimulating bacteria), the lack of expression of SAR marker genes, and the obtained results for the expression of ISR marker genes *pdf1.2* and *pr4* suggest CspD as the ISR elicitor, though this probably still needs to be confirmed using a wider panel of ISR-associated genes and under a wider range of conditions.

ROS production accompanied by an activation of the scavenging machinery is quite a common type of the rapid response of plant cells to various types of abiotic and biotic stress [36]. Analysis of expression of ROS scavengers can help to evaluate (though indirectly) the scale and dynamics of ROS production. This study showed that the CspD treatment of tobacco leaves causes ROS production, thus confirming the similar results obtained earlier for csp22 [19]. The study of expression of some common antioxidant enzymes in tobacco after the CspD treatment showed that only superoxide dismutase was overexpressed. The fact that the *sod* gene was significantly up-regulated in all experimental sets indicates an important role for this enzyme in ROS utilization under the conditions of the CspD treatment. Overexpression of the *sod* gene in upper (“systemic”) leaves demonstrates the response, which is distanced from the stimulus application point in both space and time. *Sod* expression in “systemic” leaves was measured 4 days after their treatment with CspD; however, it would be also interesting to measure its expression level in upper leaves shortly after the CspD treatment of “local” leaves in the context of putative ROS wave induction typical for systemic acquired acclimation during the abiotic stress [36]. The results of *sod* expression tests were supported biochemically: ROS assay confirmed accumulation of endogenous superoxide in “local” tobacco leaves after the CspD treatment. Interestingly, the tested superoxide dismutase was one of several isozymes, particularly CSD1, which is a Cu-Zn enzyme located mainly in the cytoplasm. According to the RNA-seq validation test, another Cu-Zn superoxide dismutase CSD2 located in chloroplasts was not overexpressed in the 6 h test, but it was not clear whether other isoenzymes are involved in the CspD-induced response.

Taking into consideration the expression maxima of *sod* and *pdf1.2* genes in the targeted gene expression tests, as well as typical time frames of available transcriptomic studies for other MAMPs [32,35], 6 h after the CspD treatment was chosen as a time point for the RNA-seq experiment. The ORA test of tobacco DEGs with log_2_FC ≥ 1.5 demonstrated the expected enrichment in GO terms related to the plant defense. However, the lack of statistically significant enriched GO terms for highly up-regulated tobacco DEGs (log_2_FC ≥ 2), probably caused by insufficient annotations of the examined genes in the reference tobacco genome, did not provide information about biological processes potentially regulated by these genes. To overcome this obstacle, the highly up-regulated tobacco DEGs were converted into much-better-annotated orthologs of a model plant *A. thaliana* and underwent the ORA test again using an AgriGO SEA web tool. The obtained list of enriched GO terms was verified by another bioinformatic web tool (geneonthology.org) and then compared with the transcriptomic data for other elicitors [32,33,34,35] using an AgriGO SEACOMPARE web tool. A comparative analysis showed that the response to light was a unique feature of the CspD-induced response. Interestingly, eight of ten non-intersecting genes, associated with the corresponding GO terms, were exclusively overexpressed in CspD-treated plants in contrast to plants exposed to other elicitors; some of these genes are involved into the photosynthesis (PHY E, PIL5, JAC), plant development (GI, ELF3, GA, AT4G32700), or gene silencing (AGO1). The remaining two genes were also expressed in plants treated with other elicitors: CSD1 was overexpressed 3.31- and 3.96-fold in plants treated with Flg22 [32] and OMV [35], respectively, and gibberellin 2-oxidase was significantly overexpressed in plants treated with all considered elicitors [32,33,34,35].

Since the CspD molecule is large enough to penetrate into an intact plant cell directly from the apoplast, it is possible that its subsequent effects on the cell, including the observed response to light, may be caused by the interaction with one or more receptors on its surface. Such interaction, on the one hand, should be quite nonspecific, so further signal transduction could trigger defense response similar to the action of flg22, OGs and OMVs. On the other hand, it should be specific enough to induce a unique response to light. One should also note that the obtained results do not indicate that the response to light is necessarily coupled with the defense pathway; it may be induced by the interaction with a receptor other than that, which triggers the defense cascade. Receptors for the cold shock proteins possessing eliciting properties were studied in relation to *N. tabacum* species of the *Solanaceae* family [28,29], but not in tobacco itself; moreover, transcriptomic studies have not been performed for these species. Therefore, it may be useful to confirm the obtained data on the resistance induction of *N. tabacum* by CspD in the transcriptomic experiments using *N. benthamiana* or *S. lycopersicum*. Furthermore, though interspecific ORA tests using orthologs are acceptable, it probably makes sense to perform such transcriptome studies in *Arabidopsis* plants to avoid any interspecific biases.

It is known that unfavorable environmental conditions, such as cold or drought, can activate ROS production in plants [37]. Interestingly, some genes from the list of CspD-induced unique GO terms, such as circadian clocks regulators GIGANTEA [38], ELF3 [39], and calcium ion-binding ANNEXIN 5 [40], have response to cold among their functions. The most over-expressed sugar transporter ERD6 gene is also involved into the cold stress response in *Arabidopsis* [41]. CspD is a cold-shock protein, an elicitor of plant defense responses. As it was found for the first time in the current study, it can induce response to light in tobacco plants (in contrast to other common MAMPs). At this point, one can suppose that CspD somehow triggers or mimics a kind of cold-priming state in tobacco plants, which then stimulates the response to light and primes the defense response. Both cold stress and exposure to light can cause excessive ROS production; however, it was shown [42] that the cold-priming and light responses are uncoupled in their influence on the defense and development responses in *Arabidopsis* plants: cold-priming stimulates plant defense at the cost of development, while the light response works in an opposite way. Thus, the exact relationship between these CspD features remains elusive and requires further examination. Since the CSD1 overexpression in upper “systemic” leaves of tobacco was first demonstrated in the current study, one of the possible next steps may be the RNA-seq of “systemic” tobacco or *Arabidopsis* leaves with the further comparison of the obtained results with the expression profile of leaves locally treated with CspD.

## 4. Materials and Methods

### 4.1. Plant Material and CspD Preparation

#### 4.1.1. CspD Isolation

Preparations of the recombinant CspD elicitor were isolated from *Escherichia coli* Shuffle and purified as described earlier [43]. The obtained protein preparations were then freeze-dried in an SP VirTis Freeze Dryer (SP Scientific, Warminster, PA, USA) and stored at −20 °C until use.

#### 4.1.2. Growing and Treatment of Tobacco Plants

Tobacco plants of a necrose-forming cv. Xanthi NN were grown up to the stage of 4–5 true leaves in individual 400-mL pots with a well-draining peat-soil mix using a Stellar-PHYTO LINE R6-L stand (AWTech, Moscow, Russia) equipped with fluorescent grow lights. The growth conditions were the following: a 16 h photoperiod, 24° C/20 °C day/night temperature, and 50–70% relative humidity.

In local expression experiments, which involved evaluation of the gene expression in CspD-treated leaves, a CspD solution was prepared in fresh distilled water at a concentration of 500 μg/mL, then applied onto the 3rd true leaves of tobacco plants (100 μL per leaf) and evenly distributed by a glass stick over the leaf surface. The 3rd true leaves of control plants were treated in the same manner but with distilled water. For the experiments involving TMV infection, both CspD-treated and control leaves were inoculated with the virus 24 h after the elicitor application in accordance with the earlier-described procedure [44]. For experiments assessing systemic expression, i.e., gene expression in untreated leaves of the same plant, the 3rd true leaves of tobacco plants were treated with the CspD solution or fresh distilled water (control) as described above. The plants were then allowed to grow for 4 days under the same growing conditions. After this period, the 4th true leaves from the next upper tier were collected for the RNA isolation and further analysis.

In all experiments, each treatment variant included 10–12 plants and was performed in two replications.

### 4.2. Gene Expression Analysis

#### 4.2.1. RNA Isolation

Total RNA was extracted from tobacco leaves using a HiPure Plant RNA Plus kit (Magen Biotechnology Co. Ltd., Guangzhou, China) following the manufacturer’s instructions; fresh leaf tissue was preliminarily homogenized in liquid nitrogen. To prevent contamination with genomic DNA during the procedure, the samples were additionally treated with DNase from the RNase Free DNase I Set (Magen Biotechnology Co. Ltd., Guangzhou, China) according to the manufacturer’s instructions. The quality of isolated RNA was evaluated spectrophotometrically using a Qubit 4 fluorimeter (Thermo Fisher Scientific, Waltham, MA, USA) and also electrophoretically in 1% agarose.

#### 4.2.2. Reverse Transcription Polymerase Chain Reaction

The reverse transcription reaction was conducted using a MMLV RT kit (Evrogen JSC, Moscow, Russia) in accordance with the manufacturer’s instructions. To assess the potential presence of genomic DNA in the cDNA samples, cDNA amplification was performed using a pair of the following primers: the forward primer 5′-AACTGGGACGATATGGAGAA-3′, which is complementary to the first exon of the beta-actin gene of tobacco; and the reverse primer 5′-GAGGCGAATCCAGAATTTAAAC-3′, which is complementary to the adjacent intron. The primers were designed based on the *Nicotiana tabacum* actin gene sequence available from GenBank (accession no. EU938079.1 *Nicotiana tabacum* actin gene, complete cds.). Following amplification, the obtained PCR products were separated via electrophoresis, and the presence of genomic DNA was indicated by the appearance of a 295-bp intron-specific product, which can be present only in an unspliced genomic DNA. Samples suspected for contamination with genomic DNA were excluded from the further study.

#### 4.2.3. Gene Expression Analysis by qPCR

Oligonucleotides used for detecting transcripts of SAR/ISR marker genes, ROS scavenger genes, and two reference genes (L25 and CP23) are listed in the Supplementary Materials (Table S7).

The reaction mixture for the qPCR assays contained SYBR Green I and was prepared using a 5× qPCRmix-HS SYBR kit (Evrogen JSC, Moscow, Russia) according to the manufacturer’s instructions. The total volume of a cDNA sample added to the reaction mix was 1 μL.

The amplification was carried out using a DT-96 thermocycler (DNA-Technology Ltd., Moscow, Russia) under universal amplification conditions including the initial denaturation step at 95 °C for 5 min, followed by 50 cycles of 95 °C for 10 s, 57 °C for 10 s, and 72 °C for 10 s. PCR was followed by a melting curve analysis within the range of 65–95 °C to minimize the potential of false positive results.

Two independent experiments were performed, each including three biological replicates.

#### 4.2.4. qPCR Data Analysis

Amplification efficiency was calculated using the Cq slope method. The relative expression of target genes was assessed according to the mathematical model proposed by Pfaffl [45]; a QGene software package v. 4.4.0 was used [46]. Results are presented as fold changes in treated samples relative to the expression of the same gene in the control sample, which was considered as 1.0.

### 4.3. RNA-Seq Analysis

#### 4.3.1. RNA Extraction, Sequencing, and Bioinformatics Processing

Leaves collected from three individual tobacco plants were treated with 500 μg/mL CspD or distilled water (control) as described in Section 4.1.1. Total RNA samples were extracted from tobacco leaves 6 h after the treatment following the protocol described in Section 4.1.2 and then sent to the Genoanalytica Ltd. (Moscow, Russia) for sequencing.

The quality and quantity of the extracted total RNA were assessed using an Agilent 2100 BioAnalyser (Agilent Technologies, Inc., Santa Clara, CA, USA) and an RNA 6000 Nano Kit of the same manufacturer according to the manufacturer’s instructions. Then, the polyA fraction of sample RNA was obtained using oligoT magnetic beads from the Dynabeads^®^ mRNA Purification Kit (Ambion, Austin, TX, USA) following the manufacturer’s instructions. Libraries for massive parallel sequencing were prepared from polyA RNA samples using a NEBNext^®^ Ultra™ II RNA Library Prep kit (New England Biolabs, Ipswich, MA, USA) according to the manufacturer’s instructions. Library concentrations were determined using a Qubit 2.0 fluorimeter (Thermo Fisher Scientific, Waltham, MA, USA) and a Qubit dsDNA HS Assay Kit of the same manufacturer. The library fragment length distribution was evaluated using an Agilent High Sensitivity DNA Kit (Agilent Technologies, Inc., Santa Clara, CA, USA). The sequencing was performed on a HiSeq1500 sequencing system (Illumina, San Diego, CA, USA).

The bioinformatics pipeline for the RNA-seq data processing included mapping with HISAT2 v.2.2.1 [47] on the GCF_000715135.1_Ntab-TN90_genomic tobacco genome assembly, counting the number of reads with HTSeq v.0.11.3 [48] in the intersection-strict mode, and a differential expression analysis with DESeq2 v.1.42.1 [49]. Only statistically significant DEGs with |log_2_FC| ≥ 1 and the adjusted *p*-value < 0.05 were used for the downstream analysis.

#### 4.3.2. Gene Descriptions and ORA Tests

Gene descriptions for the differentially expressed genes (DEGs) in tobacco were obtained from the UniProt database (www.uniprot.org). *Arabidopsis* orthologs were identified using the OMA Orthology database (https://omabrowser.org/oma/home/, accessed on 29 August 2024) and subsequently verified by the manual search in the UniProt and Ensembl (www.ensembl.org) databases. The over-representation analysis (ORA) was conducted using the Panther ORA (https://geneontology.org/, accessed on 31 August 2024) and AgriGO SEA (https://systemsbiology.cau.edu.cn/agriGOv2/specises_analysis.php?SpeciseID=1&latin=Arabidopsis_thaliana, accessed on 31 August 2024) web tools. A comparative analysis of the enriched gene ontology (GO) terms for different treatments was performed using the AgriGO SEACOMPARE tool (https://systemsbiology.cau.edu.cn/agriGOv2/c_compare.php, accessed on 15 September 2024).

### 4.4. ROS Analysis

To investigate the influence of CspD on the ROS generation in tobacco plants, a detached leaf assay was used [43,44]. Briefly, detached tobacco leaves were placed into a wet chamber. After application of CspD (500 μg/mL) or sterilized distilled water (control) drops onto leaf surface, the leaves were left at room temperature for 6 or 24 h followed by collection of the resulted diffusates from the leaf surface by a pipette for the subsequent ROS analyses.

ROS production, which resulted from the adrenaline oxidation by oxygen radicals and caused changes in a sample absorbance at 480 nm [50], was measured 45 min after adding 5 μL of a 1 mM adrenaline solution (pH 2.0, HCl) to the reaction mixture consisting of 5 μL of 0.02 M K-phosphate buffer (pH 8.2), 5 μL of either water or a SOD solution, and 35 μL of the sample or water (blank sample). The measurement was made using a Benchmark Plus spectrophotometer (Bio-Rad Inc., Tokyo, Japan). Thus, the experiment was designed as two parallel tests. The first one demonstrated the total ROS production in CspD-treated leaves via comparison of diffusates collected 6 or 24 h post-treatment with those of the SOD-free control. The second test was focused on demonstrating the specific superoxide production in CspD-treated leaves; i.e., the variant with the SOD addition was used as an additional control.

Two independent experiments were arranged, each including 10 elicitor-treated and 10 control leaves.

### 4.5. Statistical Data Treatment and Result Presentation

The statistical treatment and analysis of the obtained results were performed using a Statistica 6.0 software package (StatSoft Inc., Tulsa, OK, USA). The significance of differences (*p* < 0.05) between the means of experimental and control values was assessed using a *t*-test for independent variables. The bars in Figure 1, Figure 2 and Figure 5 represent mean values calculated from three independent experiments, each comprising 10–12 replications per treatment; error bars indicate the standard deviations (SD).

## Data Availability

The original contributions presented in this study are included in the article/Supplementary Materials. Further inquiries can be directed to the corresponding authors.

## Supplementary Materials

The following supporting information can be downloaded at https://www.mdpi.com/article/10.3390/ijms252313015/s1.
