# Supplementary material for: Transcriptomic Study of Nicotiana tabacum Treated with the Bacterial Protein CspD Reveals Some Specific Abiotic Stress Responses
Source: Int J Mol Sci. 2024 Dec 3;25(23):13015. doi: 10.3390/ijms252313015 (PMC11641646; doi:10.3390/ijms252313015)
Supplement: Supplementary file 1 [file ijms-25-13015-s001.zip › File S1.pdf]

Analysis Type:	PANTHER Overrepresentation Test (Released 20240807)
Annotation Version and Release Date:	GO Ontology database DOI: 10.5281/zenodo.12173881 Released 2024-06-17
Analyzed List:	upload_1 (Arabidopsis thaliana)
Reference List:	Arabidopsis thaliana (all genes in database)
Test Type:	FISHER
Correction:	FDR

**GO biological process complete**	**Arabidopsis thaliana - REFLIST (27475)**	**upload_1 (173)**	**Expected**	**Fold enrichment**	**raw P-value**	**FDR**
response to ozone (GO:0010193)	33	4	.21	19.25	5.39E-05	4.27E-02
chloroplast localization (GO:0019750)	36	4	.23	17.65	7.64E-05	5.29E-02
plastid localization (GO:0051644)	37	4	.23	17.17	8.53E-05	3.94E-02
response to red or far red light (GO:0009639)	222	8	1.40	5.72	8.64E-05	3.68E-02
defense response to other organism (GO:0098542)	882	19	5.55	3.42	3.28E-06	9.08E-03
defense response (GO:0006952)	1108	22	6.98	3.15	1.98E-06	1.10E-02
biological process involved in interspecies interaction between organisms (GO:0044419)	1223	21	7.70	2.73	3.06E-05	4.24E-02
response to external biotic stimulus (GO:0043207)	1209	20	7.61	2.63	7.99E-05	4.92E-02
response to other organism (GO:0051707)	1209	20	7.61	2.63	7.99E-05	4.43E-02
response to biotic stimulus (GO:0009607)	1212	20	7.63	2.62	8.27E-05	4.17E-02
response to external stimulus (GO:0009605)	1420	23	8.94	2.57	3.11E-05	3.45E-02
response to stress (GO:0006950)	3460	44	21.79	2.02	4.52E-06	8.35E-03
regulation of cellular process (GO:0050794)	4787	52	30.14	1.73	4.61E-05	4.26E-02
