# Supplementary figures and images for: Transcriptomic Study of Nicotiana tabacum Treated with the Bacterial Protein CspD Reveals Some Specific Abiotic Stress Responses
Source: Int J Mol Sci. 2024 Dec 3;25(23):13015. doi: 10.3390/ijms252313015 (PMC11641646; doi:10.3390/ijms252313015)

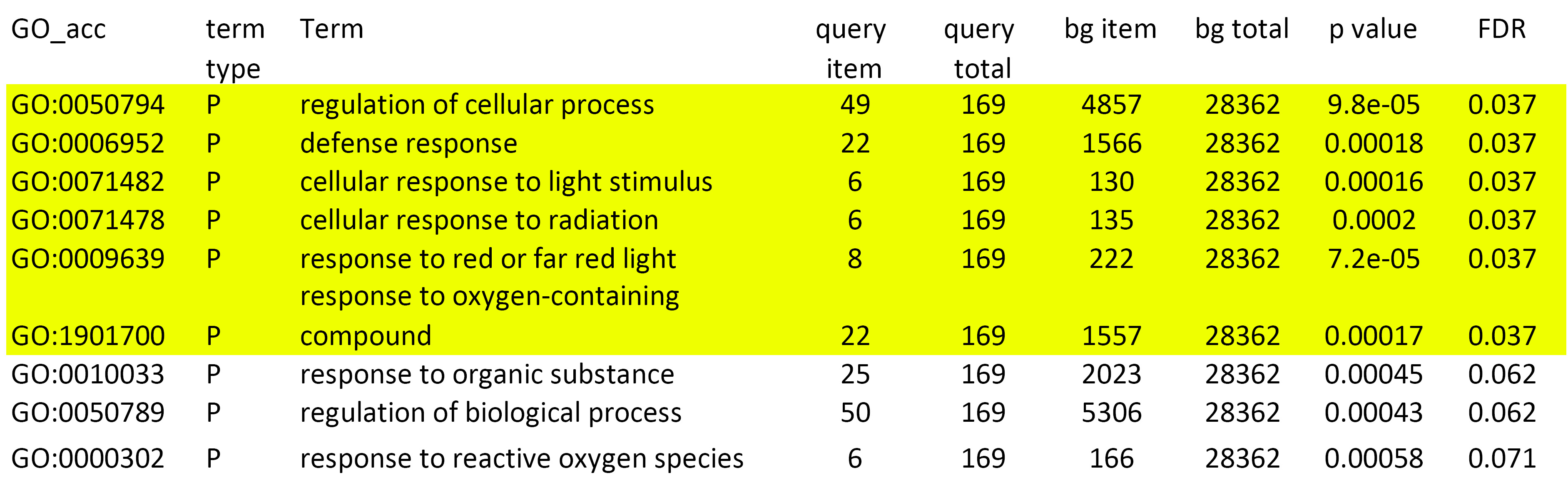

Supplement: Supplementary file 1 [file ijms-25-13015-s001.zip › File S2.jpg]
